# Algorithm for analysis of administrative pediatric cancer hospitalization data according to indication for admission

**DOI:** 10.1186/1472-6947-14-88

**Published:** 2014-10-01

**Authors:** Heidi V Russell, M Fatih Okcu, Kala Kamdar, Mona D Shah, Eugene Kim, J Michael Swint, Wenyaw Chan, Xianglin L Du, Luisa Franzini, Vivian Ho

**Affiliations:** Division of Management, Policy and Community Health, University of Texas School of Public Health, RAS E933, P.O. Box 20186, Houston, TX 77025 USA; Texas Children’s Cancer and Hematology Centers, Baylor College of Medicine, 6701 Fannin, CCC 1580.00, Houston, TX 77030 USA; Pediatric Surgery, Baylor College of Medicine, 6701 Fannin, CCC Building 1210.00, Houston, TX 77030 USA; Center for Clinical Research and Evidence-Based Medicine, University of Texas School of Medicine, Houston, TX USA; Division of Biostatistics, University of Texas School of Public Health, RAS E827, P.O. Box 20186, Houston, TX 77025 USA; Division of Epidemiology, Human Genetics and Environmental Sciences, University of Texas School of Public Health, RAS E631, P.O. Box 20186, Houston, TX 77025 USA; Baker Institute for Public Policy, Rice University, MS 40, P.O. Box 1892, Houston, TX 77251-1892 USA

**Keywords:** Cancer, Health administrative data, Healthcare utilization, Child

## Abstract

**Background:**

Childhood cancer relies heavily on inpatient hospital services to deliver tumor-directed therapy and manage toxicities. Hospitalizations have increased over the past decade, though not uniformly across childhood cancer diagnoses. Analysis of the reasons for admission of children with cancer could enhance comparison of resource use between cancers, and allow clinical practice data to be interpreted more readily. Such comparisons using nationwide data sources are difficult because of numerous subdivisions in the International Classification of Diseases Clinical Modification (ICD-9) system and inherent complexities of treatments. This study aimed to develop a systematic approach to classifying cancer-related admissions in administrative data into categories that reflected clinical practice and predicted resource use.

**Methods:**

We developed a multistep algorithm to stratify indications for childhood cancer admissions in the Kids Inpatient Databases from 2003, 2006 and 2009 into clinically meaningful categories. This algorithm assumed that primary discharge diagnoses of cancer or cytopenia were insufficient, and relied on procedure codes and secondary diagnoses in these scenarios. Clinical Classification Software developed by the Healthcare Cost and Utilization Project was first used to sort thousands of ICD-9 codes into 5 mutually exclusive diagnosis categories and 3 mutually exclusive procedure categories, and validation was performed by comparison with the ICD-9 codes in the final admission indication. Mean cost, length of stay, and costs per day were compared between categories of indication for admission.

**Results:**

A cohort of 202,995 cancer-related admissions was grouped into four categories of indication for admission: chemotherapy (N=77,791, 38%), to undergo a procedure (N=30,858, 15%), treatment for infection (N=30,380, 15%), or treatment for other toxicities (N=43,408, 21.4%). The positive predictive value for the algorithm was >95% for each category. Admissions for procedures had higher mean hospital costs, longer hospital stays, and higher costs per day compared with other admission reasons (*p*<0.001).

**Conclusions:**

This is the first description of a method for grouping indications for childhood cancer admission within an administrative dataset into clinically relevant categories. This algorithm provides a framework for more detailed analyses of pediatric hospitalization data by cancer type.

**Electronic supplementary material:**

The online version of this article (doi:10.1186/1472-6947-14-88) contains supplementary material, which is available to authorized users.

## Background

Approximately 18,000 children under 19 years old are diagnosed with a childhood cancer annually in the United States [1]. One quarter of these children have leukemia, and other common diagnoses include lymphomas, central nervous system tumors, sarcomas, and neuroblastoma. Dramatic improvements in survival over the past few decades have resulted from use of complex multimodal therapies, often associated with high risks of side effects. These medically intense treatments rely heavily on inpatient hospital resources. As the focus on healthcare utilization increases, understanding the resources required to treat children with cancer becomes more critical.

Inpatient hospital needs for childhood cancer have grown at different rates by diagnoses. Price et al. identified admissions of children with cancer as the primary discharge diagnosis and compared the mean costs in 2000 and 2009. In 2009, the overall mean cost per cancer-related admission was $40,400 and had increased by 36% from 2000 after accounting for inflation. The mean cost per Hodgkin lymphoma admission increased by 12% to $28,400, central nervous system tumor admissions increased by 61% to $39,400, and leukemia admissions by 32.3% to $55,700 during this same period [2]. Berry et al. described a 3% decrease in the prevalence of admission of children with a diagnosis of bone malignancies from 2004 to 2009, while the prevalence of admission of children with a diagnosis of acute non-lymphoid leukemia increased 10.6% [3]. Changes in the incidence of new cancer diagnoses are insufficient to explain these differences by disease [1], suggesting that changes in clinical practice play a major role. However, the complex nature of multiple cancer diagnoses, treatments, and toxicities, combined with limited research resources, means that some prioritization for studying clinical practices is required. Therefore, we wanted to identify changes in the patterns of childhood cancer admissions at a national level that may account for the differences in hospital costs and prevalence with the aim of identifying areas with the greatest increases for future studies.

The current evidence for increased use of inpatient resources is difficult to relate to oncology practice. Both Price et al. and Berry et al. used cancer diagnoses to distinguish between admissions. However, childhood cancer is a chronic condition and not, in itself, a reason for hospital admission. Most admissions for cancer are for scheduled treatment (e.g., to receive chemotherapy) or are unplanned for management of toxicities arising from cancer or its treatment [4–7]. Anticipated treatment regimens are often determined at a national cooperative group level and directly influence the frequency and severity of toxicities [8, 9]. The reason for admission is a major determinant of the type and amount of resources used [10, 11]. For example, patients admitted for chemotherapy will usually receive their pre-determined chemotherapy and be discharged quickly, whereas patients admitted for treatment of an infection are at higher risk of needing intensive care services [12].

Numerous prospective childhood cancer clinical trials have described treatment requirements and toxicities. This type of research has limited application to the study of childhood cancer hospitalizations as a whole [13, 14] because it only captures patients and events associated with individual trials. Less than half of children enroll on clinical trials when first diagnosed with cancer [15, 16] and this subset may not be wholly representative of the childhood cancer population. Adolescents have historically been underrepresented on clinical trials [15, 17] and children with pre-existing medical conditions are less likely to be eligible for participation [16]. Clinical trials must be available for the patient’s cancer, a limiting factor for patients with rare tumors or those receiving care at institutions not participating in childhood cancer clinical trials [15]. Furthermore, focusing on clinical trials will fail to capture palliative or end-of-life care, an important and costly part of medical services for childhood cancer [18]. Administrative data, or data passively collected for other indications, provides access to a more diverse population of patients and may be more accurate for studying admission patterns on a national level [19]. The Kids Inpatient Database (KID), compiled by the Healthcare Quality and Utilization Project (HCUP) and sponsored by the Agency for Healthcare Research and Quality, is a nationally representative database that samples approximately 80% of pediatric discharges from community hospitals in the United States. A version is produced every 3 years and contains between 2.5 and 3.4 million de-identified admission records from 4,839 to 5,128 hospitals in 36 to 44 states [20]. All administrative datasets pose challenges, including the structure and quality of the data and generalizability [21]. KID uses hospital admission as the primary unit of data; patients are de-identified and repeat admissions cannot be accounted for, and diagnoses and procedures are defined by billing codes. However, these limitations are balanced by the possibility of generating national hospitalization estimates.

Administrative datasets that rely on billing codes pose two challenges for studying complex diseases. The first is the assignment of the primary discharge diagnosis by coders remote from the clinical treatment team [21]. The second is the quantity of possible individual codes. The 9th version of the International Statistical Classification of Diseases and Related Health Problems (ICD-9) coding system includes approximately 12,000 diagnostic and 3,500 procedure codes. Furthermore, codes change over time. To facilitate analysis using ICD codes, HCUP developed Clinical Classification Software (CCS) which groups the thousands of ICD-9 codes into 260 mutually exclusive diagnostic groups and 231 mutually exclusive procedure groups [22]. Most of the CCS groups represent fairly homogenous diagnoses or procedures, but others combine conditions or procedures within a body system [22]. CCS groups were developed for use across a broad spectrum of potential diagnoses and may not reflect a particular clinical situation such as childhood cancer [23].

To study childhood cancer admission patterns and trends on a national level, we first sought to develop a systematic approach to classifying cancer-related admissions from administrative data into categories that reflected clinical practice. The following report describes a multi-step process for stratifying the indication for hospital admission into four categories: chemotherapy, to undergo a procedure, treatment for infection, or treatment for other toxicities. To overcome the challenges of sorting thousands of potential ICD-9 diagnostic and procedure codes, the 491 CCS groups were incorporated into the algorithm as an initial step in its development. To validate the accuracy of the CCS in correctly allowing admissions to be sorted into the four indications for admission, the ICD-9 codes in each category were individually reviewed after assignment. Finally, to test our assumption that utilization would be related to the indication for admission, mean estimates of utilization measures and rates of high intensity events were compared between admission indication categories.

## Methods

### Data source

**Figure 1:**
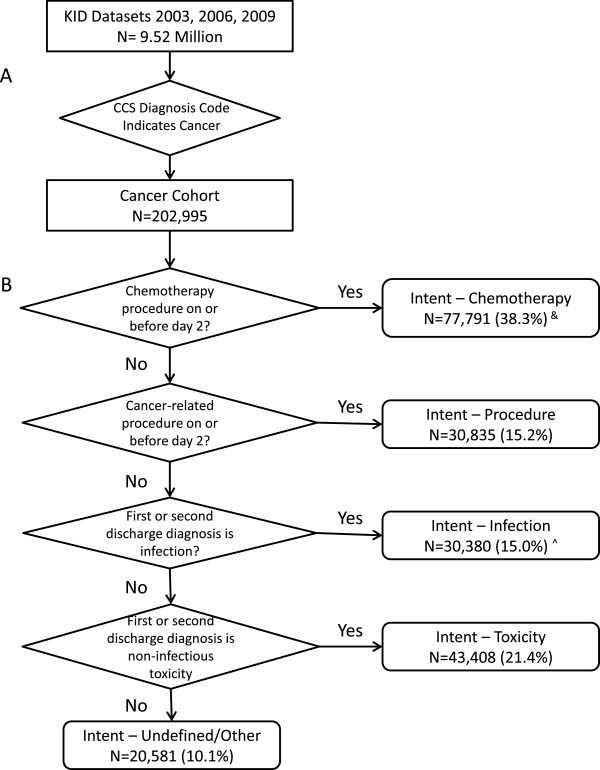
**Multi-step algorithm for stratifying childhood-cancer admissions. (A)** Identification of a cohort of cancer-related admissions from the full KID dataset. **(B)** Stratification of admissions into indications for admissions. KID indicates Kids Inpatient Database; CCS indicates Clinical Classification Software. &: Includes admissions with chemotherapy as primary procedure from states with no reported procedure dates. ^: Excludes CCS diagnostic group 237 non-infectious ICD-9 codes and non-cytopenia toxicities.

A cohort of cancer-related admissions was identified from the full KID dataset for the years 2003, 2006, and 2009 (Figure 1A). Each KID admission contained up to 15 discharge diagnoses in 2003 and 2006, and 25 discharge diagnoses in 2009. Cancer-related admissions were defined as any admission with a cancer diagnosis (CCS diagnostic groups 11 to 43) in any of the discharge diagnoses [2]. All other admissions were excluded from further analysis. In addition to diagnostic codes, each admission contained up to 15 procedural codes, basic demographic information on the patient (i.e., age, sex, healthcare payer), status of the patient upon discharge (i.e., alive or dead), and hospital location by state. States participating in HCUP elect to submit patient race/ethnicity data and the hospital day on which procedures occurred. Utilization data on each admission included length of stay (LOS) and total admission charge. This de-identified dataset was considered exempt from human subjects review by the institutional review board of the Houston branch of the University of Texas Health Science Center. The dataset was accessed after completion of a data use agreement with HCUP.

### Admission indication definitions and algorithm development

Definitions of clinically meaningful diagnostic categories, procedure categories, and admission indications were developed by consensus by a group of four pediatric oncologists (HR, FO, KK, MS) and a pediatric oncology surgeon (EK), all but one with experience in clinical processes through formal professional development. In addition, two members held clinical management roles, two with advanced training in epidemiology and one with advanced training in health economics/health services research.

Clinical experience and relevant literature supported four distinct indications for hospitalization of a child with cancer: to deliver chemotherapy (Intent-Chemotherapy), to perform a procedure (Intent-Procedure), to treat an infection (Intent-Infection), or to manage a non-infectious toxicity (Intent-Toxicity). Our approach to categorizing admissions into these four indications required two main assumptions. First, we assumed that a discharge diagnosis of cancer or cytopenia (i.e., neutropenia, anemia, thrombocytopenia) did not provide a sufficient reason for admission: cancers are chronic conditions and cytopenias are managed in an outpatient setting in the absence of other complications. Second, we assumed that when the primary discharge diagnosis did not sufficiently describe the reason for admission, the secondary discharge diagnosis would be an adequate substitute [24].We defined an indication for each admission according to a stepwise mutually exclusive algorithm (Figure 1B). The first two indications, Intent-Chemotherapy and Intent-Procedure, were identified by the performance of chemotherapy or a cancer-related procedure, respectively, during the first 2 days of hospitalization. Intent-Chemotherapy was identified first so that admissions that included minor procedures performed prior to, but not delaying, chemotherapy would be considered chemotherapy admissions rather than procedure admissions. The third and fourth indications, Intent-Infection and Intent-Toxicity, were identified from the remaining admissions by the presence of an infection or non-infectious toxicity code, respectively, as the primary discharge diagnosis or the secondary discharge diagnosis if the primary discharge diagnosis was a cancer or cytopenia. Intent-Infection admissions were identified before Intent-Toxicity to allow admissions with a primary diagnosis of cytopenia and a secondary diagnosis of infection to be classified as an Intent-Infection admission.

### CCS categories and validation

To implement the above algorithm, each primary and secondary discharge diagnostic code and each procedure code needed to be categorized into five diagnostic categories (malignancy, chemotherapy, infection, non-infectious toxicities, and other) and three procedure categories (chemotherapy, cancer procedures, and other). Chemotherapy is defined as both a procedure and diagnosis within the CCS and ICD-9 systems. This categorization was performed using the 491 CCS groups after examination by the physician group (HR, FO, KK, MS, EK) of the definition of each CCS group and the ICD-9 codes it contained. The complete categorization is presented in Additional file 1: Table S1. CCS diagnostic groups 11 through 43 were categorized as malignancy and CCS group 45 was categorized as chemotherapy. CCS diagnostic groups were categorized as infections if they included definitive or presumed infections by any microbe, fever, and/or shock. CCS diagnostic groups were categorized as non-infectious toxicities if they included toxicities arising from the cancer pathology or treatment for the cancer not previously categorized as infection, and were further sub-classified into cytopenias (CCS diagnostic groups 59, 60, and 62–64) and non-cytopenic toxicities (all others). CCS diagnostic group 237 (“Complication of device, implant or graft”) was subdivided into infections (ICD-9 999.31, 996.62, 996.67, 996.69) and non-infections (all others) as it contained large proportions of both. Diagnostic groups not related to the malignancy were categorized as “other”. Such diagnoses included pregnancy/delivery, congenital disorders, asthma, other established pediatric diagnoses, mental/behavioral disorders, and trauma. Chemotherapy procedures were limited to CCS procedure group 224. Other CCS procedure groups were categorized as cancer-related procedures if they included any procedure for the diagnosis or treatment of the malignancy or management of a treatment-related toxicity. Imaging studies and blood product transfusions were excluded. See Additional file 2 for a do-file of the CCS classification and algorithm.

To validate the accuracy of the CCS in correctly sorting admissions into admission indication categories, the ICD-9 codes for the primary and secondary discharge diagnoses and procedures occurring in the first 2 days of hospitalization were reviewed after admissions were assigned. Review of the individual codes was limited to the 90% most frequent ICD-9 codes or codes appearing more than once within that admission indication, whichever limit was achieved first. The initial sorting and review of ICD-9 codes was performed by a pediatric oncologist (HR). The clinical team then reviewed the categorization and any conflicts were resolved by discussion until consensus was obtained. The positive predictive value (PPV) of each admission indication as sorted by CCS groups was determined by calculating the percentage of the true positives (i.e., admissions with ICD-9 codes appropriate for the admission indication) within each admission category. Sensitivity was further determined by identifying admissions mis-classified by CCS groupings. We desired the algorithm to have a PPV of ≥ 95%. Because admission numbers were large, 95% confidence intervals (CI) were determined using simple asymptotic methods [25]. All analysis was performed on STATA version 11 (Stata Corp., College Station, TX, USA).

### Comparison of admission indications

We compared the admission indications by frequency of resource-intense events and utilization measures using descriptive statistics and analysis of variance. Admissions associated with hematopoietic stem cell transplantation (HCT) or requiring intensive care unit (ICU) support are resource intense events occurring frequently in childhood cancer treatment [10, 12, 26, 27]. Admissions associated with HCT were defined as those including a CCS procedure code 64 (ICD-9 41.xx). Admissions associated with ICU services were defined as those with diagnostic codes of respiratory arrest (CCS 131), cardiac arrest (CCS 107) or shock (CCS 249). Total admission costs were estimated by converting charges to costs using cost-to-charge ratios [28]. A mean cost per day for each admission was calculated as the quotient of total cost of admission over LOS. All costs were inflated to 2009 US$ using the All-Urban Consumer Price Index [29]. A *p*-value < 0.05 was considered significant.

## Results

### Overall cancer subset

We identified 202,995 admissions with a cancer diagnosis from the 2003, 2006 and 2009 KIDs. Characteristics of these admissions are presented in Table 1.

**Table 1:** **Characteristics of childhood cancer admissions**

	N = 202,995
	n (%)
Year	
2003	62,783 (30.9%)
2006	66,397 (32.7%)
2009	73,815 (36.4%)
Age at admission	
<1 y	6,722 (3.3%)
1 to 4 y	48,461 (23.9%)
5 to 9 y	43,523 (21.4%)
10 to 14 y	42,801 (21.1%)
> 15 y	60,982 (30.0%)
Gender	
Male	112,323 (55.3%)
Female	90,672 (44.67%)
Payer^	
Public&	73,973 (36.4%)
Private	113,027 (55.7%)
Other	15,678 (7.7%)
Race ^	
White	93,681 (46.2%)
Black	17,571 (8.7%)
Hispanic	37,877 (18.8%)
Other (Asian, Native American, “other”)	14,782 (7.3%)
CCS category of primary diagnosis	
Malignancy	47,853 (23.6%)
Chemotherapy	74,955 (36.9%)
Infection	30,169 (14.9%)
Non-Infectious toxicity	40,725 (20.1%)
Other	9,293 (4.6%)

&Represents both Medicaid and Medicare; ^Percentages do not sum to 100 owing to missing data. CCS indicates Clinical Classification Software.

### Admission indication

The cohort of childhood cancer admissions (Figure 1A) was sorted into admission indications using CCS diagnostic and procedure categories (Figure 1B). Review of the ICD-9 codes of the admissions in each indication validated the accuracy of this sorting process. The distribution of admissions by indication is presented in Table 2.

**Table 2:** **Positive predictive values of cancer-related admissions categorized by indication for admission**

Admission indication	CCS category of primary diagnosis					ICD-9 code			Positive predictive value (95% confidence interval)
	Malignancy N = 47,853 n (%)	Chemotherapy toxicity N = 74,955 n (%)	Infection N = 30,169 n (%)	Non-infectious toxicityN = 40,725 n (%)	Other N = 9,293 n (%)	Reviewed	True +	False +	
Intent-chemotherapy	6,985 (14.6%)	67,330 (89.8%)	1,428 (4.7%)	1,824 (4.5%)	224 (2.4%)	54,125#	53,932	193	99.6% (99.6–99.7%)
N = 77,791									
Intent-procedure	20,188 (42.2%)	1,585 (2.1%)	3,968 (13.2%)	3,258 (8.0%)	1,836 (19.8%)	45,855^	45,249	606	98.7% (98.6–98.8%)
N = 30,835									
Intent-infection	3,017 (6.3%)	249 (<1%)	23,468 (77.8%)	3,646 (9.0%)	-	28,887	28,255	632	97.8% (98.6–97.9%)
N = 30,380									
Non-infection toxicities	9,607 (20.1%)	499 (<1%)	1,305 (4.3%)	31,997 (78.6%)	-	39,485	38,565	920	97.7% (97.5–97.8%)
N = 43,408									
Undefined	8,056 (16.8%)	5,292 (7.1%)	-	-	7,233 (77.8%)				
N = 20,581									

#Admissions associated with a date of chemotherapy delivery.

^Procedures.

Intent-Chemotherapy admissions were identified first from the entire cancer cohort. The initial definition of Intent-Chemotherapy admissions identified 69,922 admissions with chemotherapy delivered in the first 2 days of hospitalization. However, only 79% of admissions with a primary CCS diagnosis of chemotherapy were included, an unexpectedly low proportion. Several states did not report dates of procedures, resulting in 10,562 (14.1%) admissions in this group without a date of chemotherapy delivery. Therefore, the Intent-Chemotherapy definition was modified to include admissions with chemotherapy delivery as the CCS group of the primary procedure, which added 7,869 admissions to Intent-Chemotherapy. Of the Intent-Chemotherapy admissions with an identifiable date of chemotherapy administration, 77.4% (N = 54,125) received chemotherapy on Day 0 of hospitalization, 16.2% (N = 11,315) on Day 1 and 5.61% (N = 3,923) on Day 2. In a few admissions (N = 193, <1%) the date of chemotherapy was recorded as negative and these were considered false positives. Chemotherapy procedure codes were included in an additional 10,583 admissions not classified as Intent-Chemotherapy. In these admissions, the median first day of chemotherapy was hospital Day 5 (range Day 3–172).

Intent-Procedure admissions were identified from the remaining cancer cohort. Both ICD-9 procedure codes and primary ICD-9 diagnostic codes (Table 2) from admissions in this indication category were reviewed. A total of 50,225 procedures were performed in the first 2 days of the 30,835 admissions identified (average 1.4 procedures per admission, range 1–14). The 10 most frequent procedures associated with these admissions are listed in Table 3. Of the 606 (1.3%) procedures not associated with cancer or treatment, 571 were appendectomy (47.xx) and 35 were umbilical vein catheterization (38.92).

**Table 3:** **Most common ICD-9 procedure codes in the first 2 hospital days of Intent-Procedure admissions**

ICD-9 procedure code	N = 50,225^ n (%)	Description
41.31	5,185 (10.3%)	Biopsy of bone marrow
38.93	4,066 (8.1%)	Venous catheterization, not elsewhere classified
01.59	3,160 (6.3%)	Other excision or destruction of lesion or tissue of brain
03.31	2,705 (5.4%)	Spinal tap
86.07	2,264 (4.5%)	Insertion of totally implantable vascular access device [VAD]
85.05	1,147 (2.3%)	Incision with removal of foreign body or device from skin and subcutaneous tissue
02.2	1,076 (2.1%)	Ventriculostomy
40.11	1,038 (2.1%)	Biopsy of lymphatic structure
40.3	827 (1.7%)	Regional lymph node excision
54.4	819 (1.6%)	Excision or destruction of peritoneal tissue

^50,225 procedures representing 1,036 individual ICD-9 procedure codes.

Intent-Infection admissions were then identified from the remaining cancer cohort and ICD-9 codes of all primary and secondary diagnoses reviewed. Cytopenias, particularly neutropenia, were the primary diagnostic codes of 12% of the Intent-Infection admissions, and malignancy accounted for another 10% (Table 2). The most frequent primary and secondary ICD-9 codes attributed to CCS groups categorized as infections are presented in Table 4. The most frequent secondary ICD-9 codes for these admissions varied slightly from those of the primary infection diagnosis (Table 4). Codes for fever, device infection, pneumonia, bacteremia, and upper respiratory tract infections were common regardless of whether the infection was the primary or secondary diagnosis. Codes listed only in primary infection diagnoses (i.e., septicemia and influenza) were the 11th and 20th (respectively) secondary diagnoses. Conversely, urinary tract infections and *C. difficile* infections were the 11th and 13th most common primary diagnoses. A notable exception was candidiasis of the mouth: despite it being the 5th most common secondary infection diagnosis, it was the 90th most common primary infection diagnosis. ICD-9 codes for infections were associated with 659 Intent-Toxicity admissions and 91 Undefined/Other admissions resulting in a sensitivity of 97.4% (95% CI, 97.2–97.6%).

**Table 4:** **Most common ICD-9 diagnosis codes observed in Intent-Infection admissions**

Intent-Infection					
CCS category of primary diagnosis of infection^			CCS category of primary diagnosis of malignancy or non-infectious toxicity Secondary CCS category of secondary diagnosis of infection ^&^		
ICD-9 diagnosis code	N = 23,468 n (%)	Description	ICD-9 diagnosis code	N = 6,912 n (%)	Description
780.6	3,316 (14.6%)	Fever and other physiologic disturbances of temperature regulation	780.61	951 (13.8%)	Fever
996.62	2,586 (11%)	Infection and inflammatory reaction due to other vascular device, implant, and graft	780.6	903 (13.1%)	Fever and other physiologic disturbances of temperature regulation
486	1,671 (7.2%)	Pneumonia, organism unspecified	790.7	585 (8.5%)	Bacteremia
780.60	1,522 (6.5%)	Fever, unspecified	486	506 (7.4%)	Pneumonia, organism unspecified
790.7	1,392 (5.9%)	Bacteremia	112.0	291 (4.2%)	Candidiasis of mouth
999.31	907 (3.9%)	Infection due to central venous catheter	996.62	285 (4.1%)	Infection and inflammatory reaction due to other vascular device, implant, and graft
038.9	744 (3.2%)	Unspecified septicemia	599.0	279 (4.1%)	Urinary tract infection, site not specified
465.9	710 (3.0%)	Acute upper respiratory infections of unspecified site	008.45	233 (3.4%)	Intestinal infection due to *Clostridium difficile*
079.99	638 (2.7%)	Unspecified viral infection	780.60	208 (3.0%)	Fever, unspecified
487.1	613 (2.6%)	Influenza with other respiratory manifestations	465.9	156 (2.3%)	Acute upper respiratory infections of unspecified site

^Includes 475 individual ICD-9 diagnosis codes.

^&^Includes 322 individual ICD-9 diagnosis codes.

The final admission indication to be identified was Intent-Toxicity. Again, ICD-9 codes of primary and secondary diagnoses were reviewed. The most common first and second ICD-9 codes in Intent-Toxicity (Table 5) demonstrated that cytopenias remained a common diagnosis even after removing those admissions with an infection as the secondary diagnosis. Of the 51% of admissions in this indication with a primary diagnosis code describing a cytopenia, the secondary diagnosis was malignancy in 79% or a second cytopenia in 15.8%. Non-infectious toxicity ICD-9 codes were associated with 491 Intent-Infection admissions and 510 Undefined/Other admissions, resulting in a sensitivity of 97.5% (95% CI, 97.3–97.6%).

**Table 5:** **Most common ICD-9 diagnosis codes observed in Intent-Toxicity admissions**

Intent-Toxicity					
CCS category of primary diagnosis of toxicity ^&^^			CCS category of primary diagnosis of malignancy or chemotherapy CCS category of secondary diagnosis of non-infection toxicity#		
ICD-9 diagnosis code	N = 33,302 n (%)	Description	ICD-9 diagnosis code	N = 10,106 n (%)	Description
288.0	8,109 (24.4%)	Neutropenia	284.8	806 (8.0%)	Other specified aplastic anemias
288.00	5,351 (16.1%)	Neutropenia NOS	331.4	734 (7.3%)	Obstructive hydrocephalus
284.8	1,920 (5.8%)	Other specified aplastic anemias	288.0	706 (7.0%)	Neutropenia
288.03	1,349 (4.1%)	Drug induced neutropenia	780.39	507 (5.0%)	Other convulsions
276.51	1,195 (3.6%)	Dehydration	287.5	437 (4.3%)	Thrombocytopenia, unspecified
276.5	658 (2.0%)	Volume depletion	284.1	330 (3.3%)	Pancytopenia
780.39	616 (1.9%)	Other convulsions	288.00	232 (2.3%)	Neutropenia NOS
996.85	433 (1.2%)	Complications of transplanted bone marrow	276.1	200 (2.0%)	Hyposmolality and/or hyponatremia
787.01	393 (1.2%)	Nausea with vomiting	285.9	195 (1.9%)	Anemia, unspecified
284.1	371 (1.1%)	Pancytopenia	518.81	192 (1.9%)	Acute respiratory failure
577.0	369 (1.1%)	Acute pancreatitis	996.85	188 (1.9%)	Complications of transplanted bone marrow

^&^Includes non-infectious ICD-9 codes from CCS diagnostic group 237.

^Includes 760 individual ICD-9 diagnostic codes.

#Includes 547 individual ICD-9 diagnostic codes.

After accounting for the four predefined indications for admission, 20,581 admissions (10.1%) remained unclassified. Only 35% of these had a primary diagnosis with a CCS category of “other”. ICD-9 V578.9 (Care involving other specified rehabilitation procedures) was the most frequent code and was associated with 509 admissions. Five of the 10 most frequent diagnostic codes were maternal/fetal in nature (ICD-9 648.91, 664.01, V300.0, 659.71, and 645.11). Almost 17% of admissions with malignancy and 7% of admissions with chemotherapy as the CCS category of the primary diagnosis remained unclassified by this algorithm. For admissions with malignancy as a primary code, malignancy was the second diagnostic code in 38.7% and “other” in 61.1%.

### Comparison of utilization by admission indication

**Figure 2:**
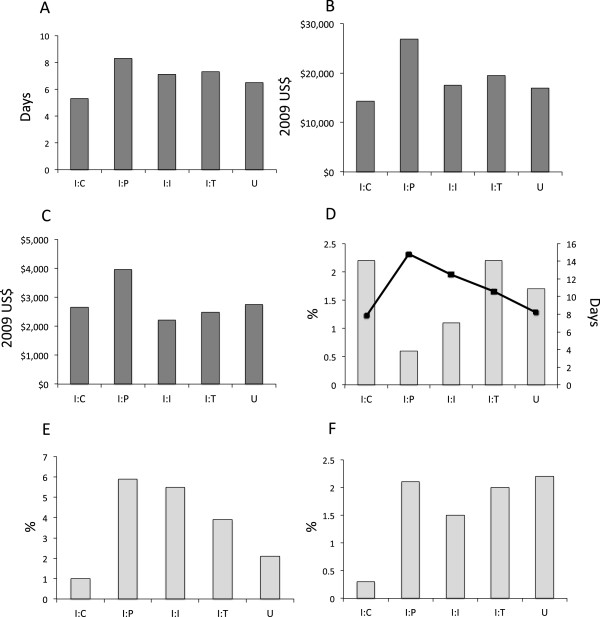
**Comparison of resource utilization measures between admission indication categories for all KID cancer-related admissions from 2003, 2006, and 2009.** Mean length of stay **(A)**, total costs per hospitalization **(B)** and cost per day **(C)** were significantly different (*p* < 0.001). Similarly, the proportion of admissions including hematopoietic stem cell transplantation **(D)**, the mean day of the transplant procedure (D, squares), admissions requiring intensive care **(E)**, and death **(F)** were significantly different (*p* < 0.001). I:C indicates Intent-Chemotherapy; I:P, Intent-Procedure; I:I, Intent-Infection; I:T, Intent-Toxicity; U, Undefined and other admissions.

Mean resource utilization, and frequency of HCT, ICU use, and death were compared across admission indications (Figure 2). Significant differences (*p* < 0.001) between measures of utilization occurred in each comparison. Intent-Procedure admissions were associated with higher mean costs of hospitalization, longer hospital stays, and higher costs per day compared with other admission indications. Rates of ICU use and death were significantly lower in Intent-Chemotherapy than in other indications. The frequency of HCT procedures was highest in Intent-Chemotherapy admissions and Intent-Toxicity admissions. However, on average, HCT occurred earlier in Intent-Chemotherapy (Day 7.8) compared with Intent-Toxicity admissions (Day 10.6).

## Discussion

This report describes a method of stratifying childhood cancer admissions into clinically meaningful reasons for admission. We used the externally developed CCS classification scheme based on ICD-9, which includes a combination of diagnostic and procedure codes, and used the secondary diagnostic codes when primary diagnostic codes were inadequate. We validated the use of the CCS classification scheme in our algorithm against the ICD-9 codes, and compared resource utilization between the admission indications. To our knowledge this is the first report to consider childhood cancer admissions in this framework.

HCUP estimated that 94,600 cancer-related admissions in 2009 cost approximately $1.9 billion [2], a substantial proportion of US pediatric healthcare expenditure. Furthermore, the healthcare resource needs of each cancer diagnosis grew at appreciably different rates during the 2000s [2, 3]. Translating such findings into strategies to improve care delivery has been slow because of a paucity of studies of patterns of inpatient care in complex diseases. Our method provides a framework for analyzing administrative data in a clinically meaningful manner.

Our method for defining a cancer-related admission cohort from the entire KID admission dataset was similar to that presented in HCUP’s 2012 report on the same topic [2], with the exception of CCS groups 44 and 45. We excluded CCS group 44 (“Neoplasms of unspecified nature or uncertain behavior”) because of the predominance of rare and benign neoplasms included in this CCS group. CCS 45 (“Maintenance chemotherapy”) was also excluded from the definition of our cancer cohort to limit the inclusion of a small subset of patients receiving chemotherapy for non-malignant disorders. We chose not to assess the sensitivity or negative predictive value of our approach in the 9.3 million non-cancer-related admissions because the sheer number of admissions was so large that both values were expected to be high.

The CCS categorization is designed and managed by HCUP and included in their data sets. This method has the distinct benefits of identifying cohorts of similar ICD-9 codes and adjustments for year to year code changes [22]. CCS groups are already included in the HCUP datasets, but the categorization software is available from HCUP and applicable to other platforms, enhancing the generalizability of this algorithm. In most scenarios of our analysis, the CCS groups adequately captured our desired diagnoses and procedures. However, there were scenarios, such as CCS diagnosis group 237, where the ICD-9 codes were distributed between categories. In the case of CCS group 237 we divided the group for better predictive value, but in other scenarios we allowed a loss of sensitivity because there were so few admissions with each ICD-9 code.

Our framework presupposes that the timing of procedures and discharge diagnoses can predict a general reason for admission, and, furthermore, that care would be more similar within each indication than between indications. Such suppositions are supported by the differences in overall utilization measures. Further, the lower rates of deaths and ICU services in admissions intended to deliver chemotherapy compared with other indications likely reflect a healthier patient population, i.e., admitted for pre-planned treatment after meeting health requirements rather than admitted in an emergency. HCT procedures, which require advanced planning and a relatively good state of patient health, occurred more frequently and earlier in the course of Intent-Chemotherapy admissions as compared with other admission indications. While more analysis is needed to understand the variation within each admission indication, the significant variation between admission indications supports our categorization of admissions.

The algorithm provides a framework for the majority of admissions. However, there remain approximately 13,000 admissions (6.4%) with either malignancy or chemotherapy toxicity as a primary discharge diagnosis that do not fit into the framework. In addition, cytopenias without a more explanatory secondary diagnosis constituted a large proportion of the Intent-Toxicity admissions. Future analyses should consider if these admissions represent an indication subset that our framework failed to capture or if they are a product of the limitations of using discharge codes [19, 21, 24, 30]. The lack of procedure dates by several states participating in KID suggests that we may have under-identified the admissions for procedures. We were able to adjust our definition of chemotherapy because of a limited number of codes, but such adjustment for over 1,000 procedure codes would be impractical and imprecise. Although some admissionsremained undefined after application of our algorithm, the admission indication categories translate to clinical practice with greater ease than grouping admissions solely by the primary diagnosis.

Patients within KID are de-identified, a structural aspect of the database which limits identification of other potentially high utilization events such as *de novo* diagnosis of cancer [13]. Because we could not link de-identified admission data with patients’ medical records or other primary sources, external validation [30] could not be performed. Furthermore, de-identification prevented the examination of multiple admissions for the same patient; therefore, no comparison between the demographics of our cancer-related admissions and an external source such as the SEER cancer registry [1, 13] was possible. KID provides the distinct advantage of a large, diverse geographic database with established methods for estimating national utilization [31], benefits which outweigh these aforementioned limitations within the context of our goal of studying patterns and trends in cancer-related inpatient resource utilization at a national level.

## Conclusions

In summary, our multi-step algorithm for categorizing childhood cancer-related admissions identifies admissions with distinct patterns of resource utilization. Future studies could use this algorithm to compare trends in indications for hospitalizations between childhood cancer diagnoses or to allow researchers to identify subsets of similar admissions for closer examination. This framework has potential for application to childhood cancer in other administrative data sets. Although this algorithm was developed for childhood cancer, similar algorithms could be useful for grouping admissions for adult malignancies or other complex conditions.

## Electronic supplementary material

Additional file 1: Table S1: Clinical Classification Software (CCS) Groups Included in Diagnosis and Procedure Categories. Table identifying which CCS groups are included in each cancer-related diagnosis and procedure category. (DOCX 45 KB)

Additional file 2: **Algorithm.** STATA do-file to categorize cancer-related admissions by CCS groups and sort them into mutually exclusive indications for admission. (DOCX 27 KB)

## Abbreviations

CCS: Clinical classification software
CI: Confidence interval
HCT: Hematopoietic stem cell transplant
HCUP: Healthcare Cost and Utilization Project
ICD-9: International statistical classification of diseases and related health problems, 9th version
ICU: Intensive care unit
KID: Kids Inpatient Database
LOS: Length of stay
PPV: Positive predictive value.
