# Supplementary material for: Algorithm for analysis of administrative pediatric cancer hospitalization data according to indication for admission
Source: BMC Med Inform Decis Mak. 2014 Oct 1;14:88. doi: 10.1186/1472-6947-14-88 (PMC4197316; doi:10.1186/1472-6947-14-88)
Supplement: Supplementary file 1 — Additional file 1: Table S1: Clinical Classification Software (CCS) Groups Included in Diagnosis and Procedure Categories. Table identifying which CCS groups are included in each cancer-related diagnosis and procedure category. (DOCX 45 KB) [file 12911_2014_850_MOESM1_ESM.docx]

**Additional file 1: Table S1** **Clinical Classification Software (CCS) Groups Included in Diagnosis and Procedure Categories**

| **Diagnosis Category: Malignancy** | | | | | |
| --- | --- | --- | --- | --- | --- |
| **CCS Diagnosis Group** | **Description** | **CCS Diagnosis Group** | **Description** | **CCS Diagnosis Group** | **Description** |
| 11 | Cancer of head and neck | 22 | Melanomas of skin | 33 | Cancer of kidney and renal pelvis |
| 12 | Cancer of esophagus | 23 | Other non-epithelial cancer of skin | 34 | Cancer of other urinary organs |
| 13 | Cancer of stomach | 24 | Cancer of breast | 35 | Cancer of brain and nervous system |
| 14 | Cancer of colon | 25 | Cancer of uterus | 36 | Cancer of thyroid |
| 15 | Cancer of rectum and anus | 26 | Cancer of cervix | 37 | Hodgkin`s disease |
| 16 | Cancer of liver and intrahepatic bile duct | 27 | Cancer of ovary | 38 | Non-Hodgkin`s lymphoma |
| 17 | Cancer of pancreas | 28 | Cancer of other female genital organs | 39 | Leukemias |
| 18 | Cancer of other GI organs; peritoneum | 29 | Cancer of prostate | 40 | Multiple myeloma |
| 19 | Cancer of bronchus; lung | 30 | Cancer of testis | 41 | Cancer; other and unspecified primary |
| 20 | Cancer; other respiratory and intrathoracic | 31 | Cancer of other male genital organs | 42 | Secondary malignancies |
| 21 | Cancer of bone and connective tissue | 32 | Cancer of bladder | 43 | Malignant neoplasm without specification of site |
| **Diagnosis Category: Chemotherapy** | | | | | |
| **CCS Diagnosis Group** | **Description** |  |  |  |  |
| 45 | Maintenance Chemotherapy |  |  |  |  |
| **Diagnosis Category: Infection** | | | | | |
| **CCS Diagnosis Group** | **Description** | **CCS Diagnosis Group** | **Description** | **CCS Diagnosis Group** | **Description** |
| 1 | Tuberculosis | 90 | Inflammation, infection of eye | 147 | Anal and rectal conditions |
| 2 | Septicemia (except in labor) | 92 | Otitis media and related conditions | 148 | Peritonitis and intestinal abscess |
| 3 | Bacterial infection, unspecified site | 97 | Peri, endo, and myocarditis, cardiomyopathy | 159 | Urinary tract infections |
| 4 | Mycoses | 122 | Pneumonia | 197 | Skin and subcutaneous tissue infections |
| 6 | Hepatitis | 123 | Influenza | 201 | Infective arthritis and osteomyelitis |
| 7 | Viral infection | 125 | Acute bronchitis | 237 | Complications of device, implant or graft |
| 8 | Other infections; including parasitic | 126 | Other upper respiratory infections | 246 | Fever of unknown origin |
| 76 | Meningitis | 133 | Other lower respiratory disease | 247 | Lymphadenitis |
| 77 | Encephalitis | 134 | Other upper respiratory disease | 248 | Gangrene |
| 78 | Other CNS infection and poliomyelitis | 135 | Intestinal infection | 249 | Shock |
| **Diagnosis Category: Non-Infectious Toxicities** | | | | | |
| **CCS Diagnosis Group** | **Description** | **CCS Diagnosis Group** | **Description** | **CCS Diagnosis Group** | **Description** |
| 49 | Diabetes mellitus without Complications | 102 | Nonspecific chest pain | 152 | Pancreatic disorders (not diabetes) |
| 50 | Diabetes mellitus with complications | 103 | Pulmonary heart disease | 153 | Gastrointestinal hemorrhage |
| 51 | Other endocrine deficiencies | 108 | Congestive heart failure; nonhypertensive | 155 | Other gastrointestinal disorders |
| 52 | Nutritional deficiencies | 109 | Acute cerebrovascular disease | 157 | Acute and unspecified renal failure |
| 55 | Fluid and electrolyte disorders | 117 | Other circulatory disease | 162 | Other diseases of bladder and urethra |
| 59^ | Deficiency and other anemia | 118 | Phlebitis; thrombophlebitis and thromboembolism | 163 | Genitourinary symptoms and ill-defined conditions |
| 60^ | Acute post-hemorrhagic anemia | 129 | Aspiration pneumonitis | 207 | Pathologic fracture |
| 62^ | Coagulation and hemorrhagic disorders | 130 | Pleurisy, pneumothorax, pulmonary collapse | 211 | Other connective tissue disease |
| 63^ | Diseases of white blood cells | 131 | Respiratory failure, insufficiency, arrest | 212 | Other bone disease and musculoskeletal deformities |
| 64^ | Other hematologic conditions | 137 | Diseases of mouth | 238 | Complications of surgical procedures or medical care |
| 81 | Other hereditary and degenerative nervous system conditions | 138 | Disorders of esophagus | 242 | Poisoning by other medications and drugs |
| 83 | Epilepsy; convulsions | 139 | Gastroduodenal ulcer | 244 | Other injuries and conditions due to external causes |
| 84 | Headache; including migraine | 140 | Gastritis and duodenitis | 250 | Nausea and vomiting |
| 95 | Other nervous system disorders | 141 | Other disorders of stomach and duodenum | 251 | Abdominal pain |
| 98 | Essential hypertension | 145 | Intestinal obstruction without hernia | 252 | Malaise and fatigue |
| 99 | Hypertension with complications and secondary hypertension | 151 | Other liver diseases | 253 | Allergic reactions |
| **Procedure Category Chemotherapy** | | | | | |
| **CCS Procedure Group** | **Description** |  |  |  |  |
| 224 | Cancer Chemotherapy |  |  |  |  |
| **Procedure Category: Cancer-Related** | | | | | |
| **CCS Procedure Group** | **Description** | **CCS Procedure Group** | **Description** | **CCS Procedure Group** | **Description** |
| 1 | Incision and Excision of CNS | 35 | Tracheoscopy and laryngoscopy with biopsy | 99 | Other OR GI Therapeutic procedures |
| 2 | Insertion, replacement, or removal of extra-cranial ventricular shunt | 36 | Lobectomy or pneumonectomy | 100 | Endoscopy and endoscopic biopsy of the urinary tract |
| 3 | Laminectomy, excision intervertebral disc | 37 | Diagnostic bronchoscopy and biopsy of bronchus | 101 | Transuretheral excision, drainage, or removal urinary obstruction |
| 4 | Diagnostic Spinal tap | 38 | Other diagnostic procedures on lung and bronchus | 103 | Nephrotomy and nephrostomy |
| 5 | Insertion of catheter or spinal stimulator and injection into spinal canal | 39 | Incision of pleura, thoracentesis, chest drainage | 104 | Nephrectomy, partial or complete |
| 7 | Other diagnostic nervous system procedures | 40 | Other diagnostic procedures of respiratory tract and mediastinum | 109 | Procedures on the urethra |
| 9 | Other OR therapeutic nervous system procedures | 42 | Other OR Rx procedures on respiratory system and mediastinum | 110 | Other diagnostic procedures of urinary tract |
| 10 | Thyroidectomy, partial or complete | 47 | Diagnostic cardiac catheterization, coronary arteriography | 112 | Other OR therapeutic procedures of urinary tract |
| 11 | Diagnostic endocrine procedures | 54 | Other vascular catheterization, not heart | 114 | Open prostatectomy |
| 12 | Other therapeutic endocrine procedures | 65 | Bone marrow biopsy | 116 | Diagnostic procedures, male genital |
| 15 | Lens and cataract procedures | 66 | Procedures on spleen | 118 | Other OR therapeutic procedures, male genital |
| 16 | Repair of retinal tear, detachment | 67 | Other therapeutic procedures, heme and lymphatic system | 119 | Oophorectomy, unilateral and bilateral |
| 17 | Destruction of lesion of retina and choroid | 71 | Gastrostomy, temporary and permanent | 120 | Other operations on ovary |
| 18 | Diagnostic procedures on eye | 72 | Colostomy, temporary and permanent | 124 | Hysterectomy, abdominal or vaginal |
| 19 | Other therapeutic procedures on eyelids, conjunctiva cornea | 73 | Ileostomy and other enterostomy | 125 | Other excision of cervix and uterus |
| 20 | Other intraocular therapeutic procedures | 74 | Gastrectomy | 130 | Other diagnostic procedures, female organs |
| 21 | Other extraocular muscle and orbit therapeutic procedures | 75 | Small bowel resection | 132 | Other OR therapeutic procedures, female organs |
| 22 | Tympanoplasty | 78 | Colorectal resection | 142 | Partial excision of bone |
| 24 | Mastoidectomy | 80 | Local excision of large intestine lesion | 157 | Amputation of lower extremity |
| 25 | Diagnostic procedures on ear | 83 | Biopsy of liver | 159 | Other procedures on musculoskeletal system |
| 26 | Other therapeutic ear procedures | 87 | Laparoscopy | 161 | Other OR therapeutic procedures on bone |
| 27 | Control of epistaxis | 89 | Exploratory laparotomy | 162 | Other OR therapeutic procedures on joints |
| 28 | Plastic procedures on nose | 90 | Excision, lysis peritoneal adhesions | 164 | Other OR therapeutic procedures on musculoskeletal system |
| 30 | Tonsillectomy and/or adenoidectomy | 92 | Other bowel diagnostic procedures | 165 | Breast biopsy and other diagnostic procedures on breast |
| 31 | Diagnostic procedures on nose, mouth and pharynx | 94 | Other OR upper GI therapeutic procedures | 166 | Lupectomy, quadrantectomy of breast |
| 33 | Other OR therapeutic procedures on nose mouth and pharynx | 96 | Other OR lower GI therapeutic procedures | 167 | Mastectomy |
| 34 | Tracheostomy, temporary or permanent | 97 | Other GI diagnostic procedures | 174 | Other non-OR therapeutic procedures on skin and breast |

^ CCS Groups included cytopenia diagnoses
