# Supplementary material for: Algorithm for analysis of administrative pediatric cancer hospitalization data according to indication for admission
Source: BMC Med Inform Decis Mak. 2014 Oct 1;14:88. doi: 10.1186/1472-6947-14-88 (PMC4197316; doi:10.1186/1472-6947-14-88)
Supplement: Supplementary file 2 — Additional file 2: Algorithm. STATA do-file to categorize cancer-related admissions by CCS groups and sort them into mutually exclusive indications for admission. (DOCX 27 KB) [file 12911_2014_850_MOESM2_ESM.docx]

**Additional file 2** **Algorithm.**

** The following do-file demonstrates the categorization of clinical classification software (CCS),

** and how these categories are applied to sort cancer-related admissions into

** mutually exclusive admission indications

** The variables included in the do-file are included in and defined by the KIDs dataset

** (http://hcup-us.ahrq.gov)

** unless expressly defined below.

*********************************************************************

** Define the categories (malignancy, chemotherapy, infection, toxicity, or other) **

** of the primary and secondary diagnosis CCS codes for all admissions **

*********************************************************************

** The primary diagnosis and secondary CCS diagnosis group is categorized for each admission

#delimit

foreach i of numlist 1 2 {;

generate DXcode_group_`i' = 0 ;

};

** Malignancy diagnosis category

#delimit

foreach i of numlist 1 2 {;

replace DXcode_group_`i' = 1 if (DXCCS`i' >=11 & DXCCS`i' <44);

};

#delimit cr

** Chemotherapy diagnosis category

#delimit

foreach i of numlist 1 2 {;

replace DXcode_group_`i' = 2 if DXCCS`i'==45;

};

#delimit cr

** Before defining infections and toxicities must devide CCS diagnosis group 237 into

** infection and non-infection by specific ICD-9 codes

#delimit

foreach i of numlist 1 2 {;

generate DX237_infection_`i'= 0;

};

#delimit cr

#delimit

foreach i of numlist 1 2 {;

replace DX237_infection_`i' = 1 if (DX`i' =="99662") | (DX`i' =="99931") |

(DX`i' =="99663") | (DX`i' =="99669") | (DX`i' =="99667");

};

#delimit cr

** Infection diagnosis category

#delimit

foreach i of numlist 1 2 {;

replace DXcode_group_`i' = 3 if (DXCCS`i' >=1 & DXCCS`i' <=4) | (DXCCS`i' >=6 & DXCCS`i' <=8) |

(DXCCS`i' >=76 & DXCCS`i' <=78) | (DXCCS`i' ==90 ) | (DXCCS`i' == 92 ) |

(DXCCS`i' == 97) |(DXCCS`i' >=122 & DXCCS`i' <=123) | (

DXCCS`i' >=125 & DXCCS`i' <=126) |(DXCCS`i' >=133 & DXCCS`i' <=135) |

(DXCCS`i' ==147 ) |(DXCCS`i' ==148 ) | (DXCCS`i' == 159) |

(DXCCS`i' == 197) | (DXCCS`i' ==201) | (DX237_infection_`i' == 1) |

(DXCCS`i' >= 246 & DXCCS`i' <= 249 );

};

#delimit cr

** Toxicity diagnosis category

#delimit

foreach i of numlist 1 2 {;

replace DXcode_group_`i' = 4 if (DXCCS`i' >=49 & DXCCS`i' <= 52) | (DXCCS`i' ==55) |

(DXCCS`i' >=49 & DXCCS`i' <= 52) |(DXCCS`i' >=59 & DXCCS`i' <= 60) |

(DXCCS`i' >=62 & DXCCS`i' <= 64) | (DXCCS`i' ==95) |(DXCCS`i' ==98) |

(DXCCS`i' ==99) | (DXCCS`i' ==102) | (DXCCS`i' >=137 & DXCCS`i' <= 141) |

(DXCCS`i' ==145) | (DXCCS`i' == 152) | (DXCCS`i' == 155) | (DXCCS`i' == 157) |

(DXCCS`i' == 162) | (DXCCS`i' == 207) | (DXCCS`i' == 212) | (DXCCS`i' == 238) |

(DXCCS`i' == 242) | (DXCCS`i' >=250 & DXCCS`i' <= 253) |

(DXCCS`i' == 237 & DX237_infection_`i'==0) | (DXCCS`i' == 131) | (DXCCS`i' == 83) |

(DXCCS`i' == 130) |(DXCCS`i' == 151) | (DXCCS`i' == 163) | (DXCCS`i' == 244) |

(DXCCS`i' == 118) | (DXCCS`i' == 153) |(DXCCS`i' == 109) | (DXCCS`i' == 103) |

(DXCCS`i' == 211) |(DXCCS`i' == 109) | (DXCCS`i' == 103) | (DXCCS`i' == 211) |

(DXCCS`i' == 129) | (DXCCS`i' == 84) | (DXCCS`i' == 81) | (DXCCS`i' == 108) |

(DXCCS`i' == 117);

};

#delimit cr

** Identify the CCS diagnosis groups considered cytopenias (anemia, thrombocytopenia, leukopenias)

#delimit

foreach i of numlist 1 2 {;

generate DXcode_cytopenia_`i'= 0;

};

#delimit cr

#delimit

foreach i of numlist 1 2 {;

replace DXcode_cytopenia_`i' = 1 if (DXCCS`i' >=59 & DXCCS`i' <= 60) |

(DXCCS`i' >=62 & DXCCS`i' <= 64);

};

#delimit cr

************************************************************************

** Categorize the procedure codes into chemotherapy, cancer-related and "other" **

***********************************************************************

** All 15 potential procedure codes are categorized

#delimit

foreach i of numlist 1 2 3 4 5 6 7 8 9 10 11 12 13 14 15 {;

generate PRcode_group_`i' = 0 ;

};

#delimit cr

** Chemotherapy procedures

#delimit

foreach i of numlist 1 2 3 4 5 6 7 8 9 10 11 12 13 14 15 {;

replace PRcode_group_`i' = 1 if PRCCS`i'==224;

};

#delimit cr

** Day chemotherapy was given

generate chemo_day = .

#delimit

foreach i of numlist 1 2 3 4 5 6 7 8 9 10 11 12 13 14 15 {;

replace chemo_day = PRDAY`i' if chemo_day==. & PRCCS`i'==224;

};

#delimit cr

** Cancer-related procedures

#delimit

foreach i of numlist 1 2 3 4 5 6 7 8 9 10 11 12 13 14 15 {;

replace PRcode_group_`i'= 2 if (PRCCS`i'>=1 & PRCCS`i'<=5) |

(PRCCS`i' ==7) | (PRCCS`i' >=9 & PRCCS`i'<=12) |

(PRCCS`i' >=15 & PRCCS`i'<=22) | (PRCCS`i' >=24 & PRCCS`i'<=28) |

(PRCCS`i' >=30 & PRCCS`i'<=31) | (PRCCS`i' >=33 & PRCCS`i'<=40) |

(PRCCS`i'==42) | (PRCCS`i' ==47 ) | (PRCCS`i'==54 ) | (PRCCS`i'==65) |

(PRCCS`i'>=66 & PRCCS`i'<=67) | (PRCCS`i'>=71 & PRCCS`i'<=75) |

(PRCCS`i'==78) | (PRCCS`i'==80) | (PRCCS`i'==83 ) | (PRCCS`i'==87) |

(PRCCS`i'>=89 & PRCCS`i'<=90) | (PRCCS`i'==92) |

(PRCCS`i'==94) | (PRCCS`i'>=96 & PRCCS`i'<=97) |

(PRCCS`i'>=99 & PRCCS`i'<=100) | (PRCCS`i'==101) |

(PRCCS`i'>=103 & PRCCS`i'<=104 ) | (PRCCS`i'>=109 & PRCCS`i'<=110) |

(PRCCS`i'==112) | (PRCCS`i'==114 ) |

(PRCCS`i'==116 ) | (PRCCS`i'>=118 & PRCCS`i'<=120 ) |

(PRCCS`i'>=124 & PRCCS`i'<=125 ) | (PRCCS`i'==130 ) |

(PRCCS`i'==132 ) | (PRCCS`i'==142 ) | (PRCCS`i'==157 ) |

(PRCCS`i'==159 ) | (PRCCS`i'>=161 & PRCCS`i'<=162 ) |

(PRCCS`i'>=164 & PRCCS`i'<=167 ) | (PRCCS`i'==174 );

};

#delimit cr

***************************************************************

** Use CCS diagnosis and procedure codes to define admission intentions **

***************************************************************

***********************

** Intent-Chemotherapy **

***********************

generate ADMIT_CHEMO = 0

generate INTENT = 0

** For admissions where the date of the procedure is reported, identify admissions with a

** chemotherapy procedure performed on or before day 2

#delimit

foreach i of numlist 1 2 3 4 5 6 7 8 9 10 11 12 13 14 15 {;

replace ADMIT_CHEMO = 1 if (PRcode_group_`i'==1 & PRDAY`i'<=2);

};

#delimit cr

** For admissions where the date of the procedure is not reported, identify admissions with

** a primary diagnosis of chemotherapy and a primary procedure of chemotherapy

replace ADMIT_CHEMO = 1 if DXcode_group_1==2 & PRcode_group_1 == 1 & chemo_day==.

replace INTENT= 1 if ADMIT_CHEMO==1

********************

** Intent-Procedure **

********************

generate ADMIT_SURG = 0

#delimit

foreach i of numlist 1 2 3 4 5 6 7 8 9 10 11 12 13 14 15 {;

replace ADMIT_SURG = 1 if (PRcode_group_`i'==2 & PRDAY`i'<=2);

};

#delimit cr

replace INTENT=2 if ADMIT_SURG ==1 & ADMIT_CHEMO==0

******************

** Intent-Infection **

******************

generate INFX = 0

#delimit

foreach i of numlist 1 2 {;

replace INFX = 1 if DXcode_group_`i'==3;

};

#delimit cr

generate ADMIT_INFX = 0

replace ADMIT_INFX =1 if INFX == 1 & ADMIT_CHEMO==0 & ADMIT_SURG == 0 & DXcode_group_1 !=0

** Admissions with non-cytopenic toxicities as primary diagnosis are removed so they can be

** included in toxicity admission

replace ADMIT_INFX = 0 if ADMIT_INFX ==1 & DXcode_cytopenia_1 == 0 & DXcode_group_1==4

replace INTENT=3 if ADMIT_INFX==1 & ADMIT_CHEMO==0 & ADMIT_SURG == 0

*****************

** Intent-Toxicity **

*****************

generate TOX = 0

#delimit

foreach i of numlist 1 2 {;

replace TOX = 1 if DXcode_group_`i'==4;

};

#delimit cr

generate ADMIT_TOX = 0

replace ADMIT_TOX =1 if TOX==1 & ADMIT_CHEMO==0 & ADMIT_SURG==0 & ADMIT_INFX==0 & DXcode_group_1 !=0

replace INTENT=4 if ADMIT_TOX==1 & ADMIT_CHEMO==0 & ADMIT_SURG==0 & ADMIT_INFX==0

label define intentgroup 0 "other" 1 "Intent-chemotherapy" 2 "Intent-procedure" 3 "Intent-infection"///

4 "Intent-toxicity"

label values INTENT intentgroup
